# Incidence and concomitant chondral injuries in a consecutive cohort of primary traumatic patellar dislocations examined with sub-acute MRI

**DOI:** 10.1007/s00264-023-05707-y

**Published:** 2023-02-07

**Authors:** Anders Isacsson, Ola Olsson, Martin Englund, Richard B. Frobell

**Affiliations:** 1 Department of Clinical Sciences Lund, Faculty of Medicine, Lund University, Lund, Sweden; 2 Department of Orthopedics, Helsingborg Hospital, Charlotte Yhléns gata 10, 251 87 Helsingborg, Region Skane Sweden; 3 Department of Clinical Sciences Lund, Orthopedics, Clinical Epidemiology Unit, Faculty of Medicine, Lund University, Lund, Sweden

**Keywords:** Patellar dislocation, Epidemiology, Sports, Osteochondral injury

## Abstract

**Purpose:**

To present age- and sex-specific cumulative annual incidences of primary traumatic lateral patellar dislocation (LPD) and to detail patient characteristics and concomitant chondral injuries including osteochondral fractures, as visualized on magnetic resonance imaging (MRI), in a large consecutive cohort of knee-injured individuals.

**Methods:**

Data on primary traumatic lateral patellar dislocations were collected from a large consecutive cohort of knee injuries examined with sub-acute MRI in a single centre with a well-defined catchment area. Annual incidences for different age-groups in relation to gender were calculated together with the risk of concomitant chondral and osteochondral injury, during sports and in general.

**Results:**

A total of 184 primary patellar dislocations were identified in the cohort of 1145 acute knee injuries (*n*=175) and surgical records (*n*=9). Knee MRI was performed within a median of six days of injury. Median age of patients with primary LPD was 16 years (interquartile range, 14–21; range, 9–47) and 41% were females. Males were significantly older than females at the time of injury (median age 17 vs. 15, *P* = 0.021) and sustained their primary LPD during sports more often than females (65 vs. 40%, *P* < 0.001). Primary LPD occurred most frequently at the age of 13 to 15 years where the annual incidence was 125 (95% CI, 96–160) per 100,000 persons. The overall annual incidence of primary LPD was 14 (95% CI, 12–16) per 100,000 persons, with a predominance of males versus females (17 vs. 11, *P* = 0.01). Concomitant lesions to joint surfaces were displayed on MRI or during surgery in 75 (43%) knees. Osteochondral fractures were seen in 32 knees (18%). We found no statistically significant difference in the risk of osteochondral fracture between those injured during sports or during leisure activity (14 vs. 24%, *P* = 0.08).

**Conclusions:**

The annual incidence of first-time patellar dislocation was found to be 14 per 100,000 individuals with the highest incidence found among those aged 13–15 years. Primary LPD was more common among males and was sustained during sports activity in 55% of the cases. Associated injuries to the chondral surfaces should be expected in 43% of knees with primary LPD where 18% represent osteochondral fractures.

## Introduction

Lateral patellar dislocation (LPD) is reported to be the most common cause of traumatic haemarthrosis of the knee in a paediatric population [1] and is estimated to account for some 20% of all soft tissue injuries to the knee [2, 3] in the general population. This injury has the potential to be associated with damage to the cartilage and may result in subsequent instability and higher risk of osteoarthritis in the longer term [4–6]. When an osteochondral fracture is identified in the acute phase, worse outcome may be expected [7]. In such cases, early surgery, focused on reinsertion of osteochondral fragments, has been recommended which stresses the need for early detection and a correct diagnosis [8–10].

Still, as many as 50% of all acute LPD have been reported to be clinically misdiagnosed in the acute setting [11]. While LPD may be difficult to distinguish clinically from other acute knee injuries, magnetic resonance imaging (MRI) findings are characteristic with typical post traumatic bone marrow lesion (BML) patterns located in the anterolateral portion of the lateral femoral condyle and medial patella [12–17]. Use of MRI in the acute phase after knee injury may thus increase the diagnostic precision and detect injuries that may need early surgical intervention [18].

The incidence of LPD has been studied in several different populations and age-groups [1, 19–26]. However, most of these reports are based on clinically diagnosed injuries rather than based on MRI findings, and it is possible that reported incidences are underestimates.

The purpose of this study was to present age- and sex-specific cumulative annual incidences of primary LPD. Data was retrieved from a consecutively collected cohort of 1145 patients, with acute knee injury and traumatic haemarthrosis, who all underwent subacute MRI within six weeks of the injury. Furthermore, the aim was to detail patient characteristics and concomitant chondral injuries including osteochondral fractures, as visualized on MRI. Potentially, MRI-derived diagnosis of primary LPD in a large consecutive cohort of knee-injured individuals may provide an important addition to the knowledge on the topic in relation to recent and earlier reports.

## Methods

The study was approved by the local institutional review board and was conducted at a university-affiliated emergency hospital, serving the pediatric as well as the adult population in a well-defined geographical region.

Between 1 January 2002 and 29 February 2008, 1145 consecutive individual knees with acute trauma and rapid effusion indicating traumatic haemarthrosis underwent a sub-acute MRI and were prospectively registered at the hospital as formerly reported [3]. The recruiting hospital was the only referral center in the catchment area and the only hospital or healthcare facility dealing with acute adult and pediatric trauma or sports injuries in the relevant region. Physiotherapists and primary care physicians in the area were informed of the study beforehand and were encouraged to refer all patients with acute knee trauma to either the emergency department or a sub-acute knee specialist out-patient clinic. Based on the MRI reports, 199 LPD were identified. Following collection of baseline characteristics from medical records according to predefined protocols (including clinical diagnosis at presentation, activity at injury, previous injury to either knee and estimation of Tegner activity score [27]), 168 consecutive knees with an acute first-time LPD, as visualized on MRI, were identified. Initial clinical evaluation was made at a median of one (range 0–31) day from injury and MRI was performed within a median of six (1–39) days of injury. Earlier history of patellofemoral pain or subluxation was accepted if it was obvious in the patients’ medical records that no frank dislocation had occurred. An additional seven knees were found to have a documented primary LPD during the study period, but the MRI was performed after a secondary event. Knees with a history of previous patellofemoral dislocation or surgery for patellofemoral instability outside the study period were excluded. Hence, 175 knees with primary LPD during the study period were included from the main cohort.

**Fig. 1:**
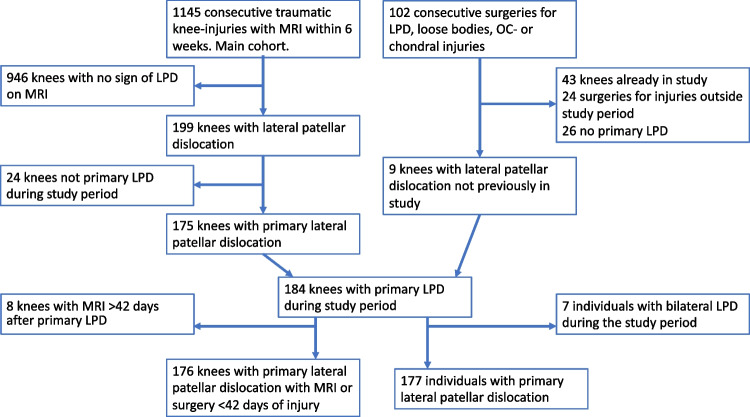
Flow chart of patient selection. LPD, lateral patellar dislocation. MRI, magnetic resonance tomography. OC, osteochondral

Some patients with a positive finding on the plain radiography, indicating acute osteochondral injury, were scheduled for surgery without prior MRI. At the time, arthroscopic surgery was only offered at two hospitals in the geographical region serving the studied population. All surgical procedures are noted together with the patients’ unique personal identification number. We scrutinized the surgical records for patients being operated on during the study period under any of the following codes: LPD (S83.0), chondral injury (S83.3) or loose body (M23.4), fixation of articular surface fragments (NGF21, NGF22), excision of articular surface fragments or loose body (diagnosis codes S83.0, S83.3, and M23.4 according to ICD10 and surgery codes NGF 21, 22, 31, 41 according to NCSP 96, NOMESCO Classification of Surgical Procedures) and found nine knees with primary LPD that were not included in the consecutive MRI cohort. Seven individuals suffered a contralateral LPD during the study period. The second knee affected was excluded from incidence calculations. Therefore, the final study population comprised 184 knees with primary LPD in 177 individuals. Eight of these knees were examined with MRI more than 42 days after injury and were excluded from calculations on concomitant intra-articular injuries (Fig. 1).

Plain radiographs were obtained as part of the initial clinical routine in 124 of the 184 knees.

### Magnetic resonance images and their evaluation

As previously described [3, 11], two different MRI scanners, a 1,5 T scanner (Gyroscan, Intera, Philips, Eindhoven, the Netherlands) and a 1,0 T scanner (Impact, Siemens, Erlangen, Germany), were used with a circular polarized coil throughout the study. T2-weighted turbospin-echo (tSEPdT2) and turbo short-tau inversion recovery (tSTIRT2) sequences were obtained in the coronal and sagittal plane. All MR images were consecutively evaluated by one of two experienced musculoskeletal radiologists in the normal clinical setting. The two radiologists did not report any relevant difference in image quality between the two scanners.

**Fig. 2:**
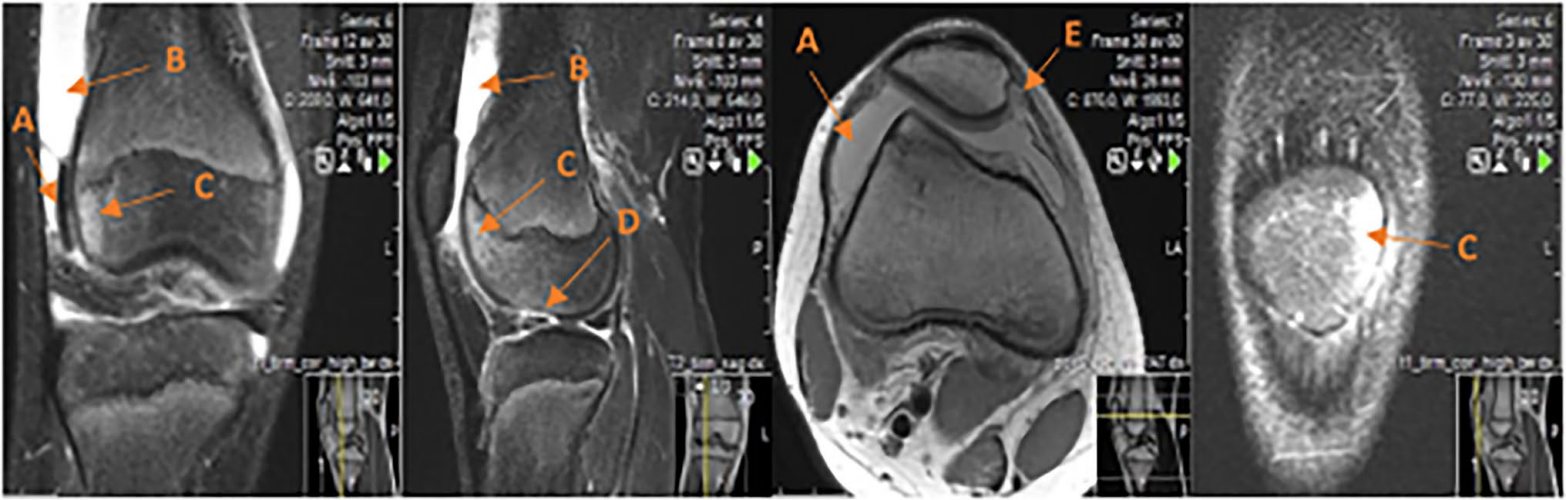
A loose chondral fragment (**A**) in the lateral recess of a 13-year-old boy following lateral patellar dislocation sustained during leisure activity with large effusion (**B**) and typical bone marrow edema (**C**). The injury site is on the lateral femoral condyle (**D**) and there is a small avulsion fracture on the medial border of the patella (**E**)

Information about structural lesions (including BML, ligamentous injury, meniscal injury, chondral injuries, osteochondral fractures, impaction fractures) and their location as well as typical signs of LPD were classified according to the recommendations published by Khanna et al. [28] and described in the analysis protocol. A recent LPD was determined by the presence of (1) knee effusion and (2) typical post-traumatic BML distribution located in the medial patella and/or the lateral femoral condyle (Fig. 2).

### Statistics and incidence calculations

Official population statistics of Sweden, held by Statistics Sweden, delivers population data for each gender divided into 1-year age groups and sex from the last of December each year. The total population within the catchment area of the hospital was 155,870 individuals for 2002. In October 2004, the orthopaedic emergency care catchment area was increased by 81,549, resulting in a total population within the catchment area of 246,999 individuals at the end of 2007. Incidences were calculated as the number of primary dislocations per 100,000 person-years for those at risk annually. For incidence calculations, the second LPD in those with bilateral injuries was excluded.

Statistical analyses were performed using SPSS version 28 for Mac (IBM Corp, Armonk, NY, USA), confidence intervals were determined using Epitools (Sergeant, ESG, 2018. Epitools Epidemiological Calculators. Ausvet), and comparisons between proportions were made in MedCalc (MedCalc Software, Ostend, Belgium). Continuous variables were expressed as medians and interquartile range (IQR). For normally distributed continuous data, the Mann-Whitney *U*-test was used in comparisons between groups. Comparison of binomial data was performed with chi-square test. Confidence intervals were determined using the Clopper-Pearson exact method and comparisons between proportions were made using the Fisher’s exact test. A two-tailed *P* value < 0.05 was considered as statistically significant.

## Results

### Patient characteristics

**Fig. 3:**
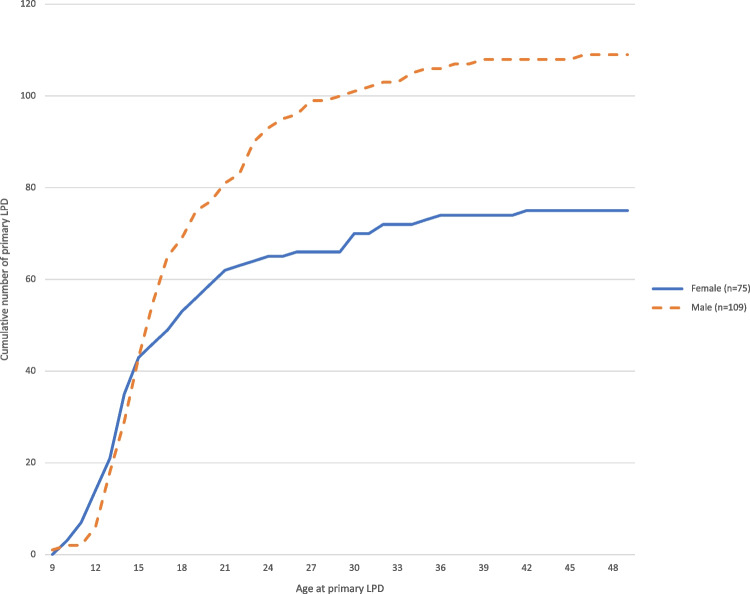
Cumulative numbers of primary traumatic lateral patellar dislocations (LPD) at different ages

Primary LPD occurred at a median age of 16 years (interquartile range (IQR), 14–21, Table 1) and was more common among males than females (59 vs. 41%, *P* = 0.015). Ninety-nine percent of primary LPD occurred before the age of 40 whereas 55% occurred before the age of 17 (Fig. 3). Males were significantly older than females at the time of primary LPD (median age 17 [IQR, 15–22] and 15 [IQR, 13–20] years, respectively, *P* = 0.021). All but one individual who sustained a primary contralateral LPD during the study period were under the age of 16 at the time of the index injury. The absolute risk of sustaining a primary contralateral LPD during the study period was 7% for those under the age of 16 (*n*=6/83) compared to only 1% for those who were 16 years or older (*n*=1/94) at their first dislocation.

**Table 1:** Demographic characteristics, primary patellar dislocation ^a^

	All	Female	Male	*P* value
Individual knees, *n*	184	75 (41%)	109 (59%)	0.015
Age at injury, years	16 (14–21;9–47)	15 (13–20; 10–43)	17 (15–22; 9–47)	0.021
Time to MRI, days	6 (3–10;1–39)	6 (4–13; 1–34)	5 (3–9; 1–39)	0.197
Right knee, *n*	93 (51%)	38 (51%)	55 (50%)	
Injury at sports, *n*	101 (55%)	30 (40%)	71 (65%)	<0.001
Annual incidences per 100,000 persons^b^, n*				
All ages (*n*=177)	14 (12–16)	11 (9–14)	17 (14–20)	0.010
10–12 years (*n*=19)	41 (24–63)	61 (34–103)	21 (7–48)	
13–15 years (*n*=63)	125 (96–160)	114 (76–165)	135 (94–187)	
16–18 years (*n*=33)	69 (47–97)	39 (18–74)	97 (62–144)	
19–21 years (*n*=21)	49 (30–75)	43 (19–81)	56 (29–97)	
22–24 years (*n*=15)	38 (21–62)	15 (3–45)	60 (31–104)	
Knees with any lesion on chondral surfaces^c^, *n*				
All locations	75 (43%)	29 (41%)	46 (44%)	0.696
Patella	33 (19%)	15 (21%)	18 (17%)	
Femur	29 (16%)	8 (11%)	21 (20%)	
Combined	13 (7%)	6 (8%)	7 (7%)	
No lesion	101 (57%)	42 (59%)	59 (56%)	
*Chondral lesion at sports*	*37 (49%)*	*9 (31%)*	*28 (61%)*	0.012
Knees with osteochondral fractures^c^, *n*				
All locations	32 (18%)	15 (21%)	17 (16%)	0.405
Patella	17 (10%)	11 (15%)	6 (6%)	
Femoral	13 (7%)	3 (4%)	10 (10%)	
Combined	2 (1%)	1 (1%)	1 (1%)	
No OC fracture	144 (82%)	56 (79%)	88 (84%)	
*OC fracture at sports*	*13 (41%)*	*4 (27%)*	*9 (53%)*	0.131

^a^Data are provided as medians (interquartile range; range), *n* (%), or cumulative annual incidence per 100,000 persons (95% CI)

^b^Contralateral knees with lateral patellar dislocation (*n*=7) are excluded for incidence calculations (*n*=177; 72 female, 105 male)

^c^Osteochondral fractures, impaction fractures, and pure chondral damage as seen on MRI or during surgery within 42 days of primary LPD (*n*=176; 71 female, 105 male). *OC* osteochondral, *MRI* magnetic resonance tomography, *LPD* lateral patellar dislocation

Fifty-five percent of all primary LPD were sustained during sporting activity with more such injury mechanisms among males than females (65 vs. 40%, *P* < 0.001). Soccer was the most common sport at injury for both genders, followed by gymnastics, martial arts, and alpine skiing (Table 2).

**Table 2:** Activity during primary lateral patellar dislocation

		All (*n*=184)	Women (*n*=75)	Men (*n*=109)	
	All sports	101 (55%)	30 (40%)	71 (65%)	*P* < 0.001
Sports	Soccer	48 (48%)	9 (30%)	39 (55%)	
	Gymnastics	12 (12%)	7 (23%)	5 (7%)	
	Martial arts	9 (9%)	1 (3%)	8 (11%)	
	Alpine skiing	7 (7%)	4 (13%)	3 (4%)	
	Ice hockey	5 (5%)	0	5 (7%)	
	Other sports	20 (20%)	9 (30%)	11 (15%)	
Leisure or non-specified activity		83 (45%)	45 (60%)	38 (35%)	

### Incidences

**Fig. 4:**
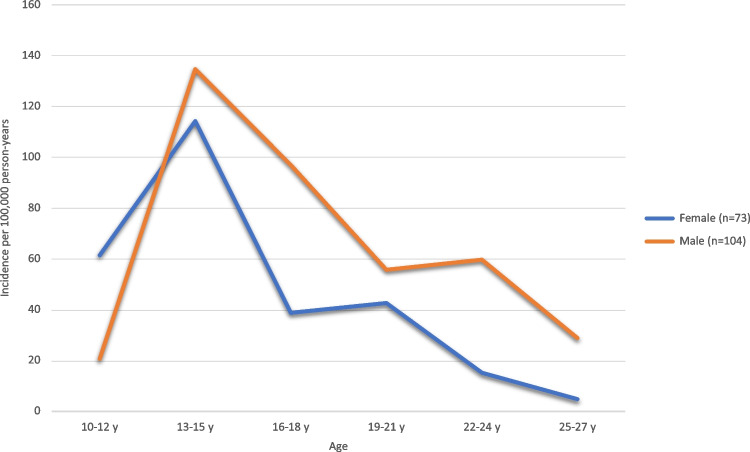
Cumulative annual incidences per 100,000 persons. 3-year age-groups

The overall annual incidence of LPD in the population was 14 (95% CI, 12–16) per 100,000 person-years, or 11 (9–14) and 17 (14–20) for females and males respectively (*P* = 0.010, Table 1). A peak of annual incidences for both females and males occurred between the ages of 13 and 15 years (114 [76–165] and 135 [94–187] per 100,000 person-years, respectively) and declined gradually over older ages (Fig. 4).

### Concomitant chondral and osteochondral lesions as visualized on MRI or during surgery in the acute period

**Fig. 5:**
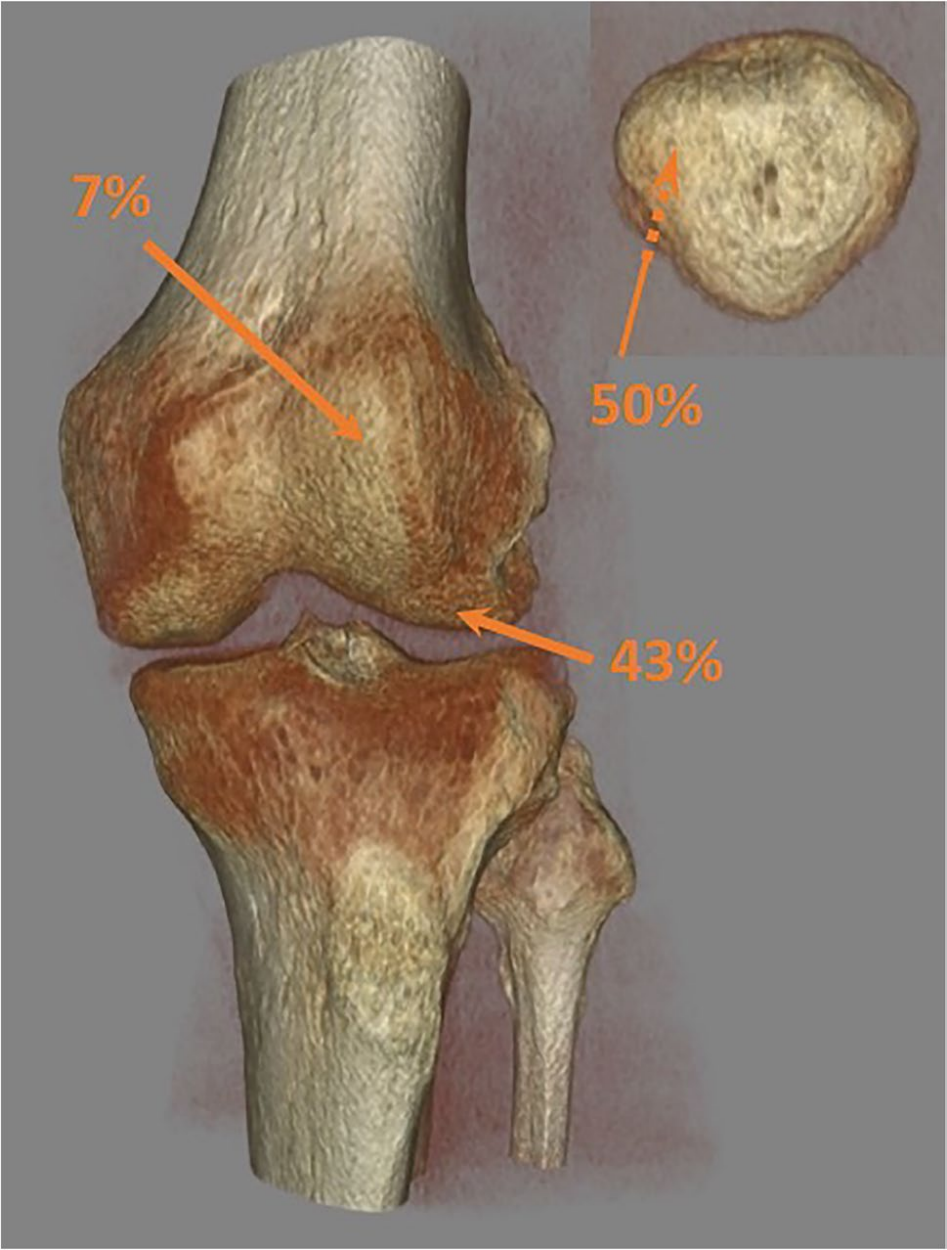
Distribution of 113 chondral injuries (chondral lesions [*n*=69], osteochondral fractures [*n*=34], or impaction fractures [*n*=10]) in 75 (43%) knees with concomitant injuries to the chondral surfaces

Magnetic resonance images were obtained in 171 knees and five knees were scheduled for surgery without prior MRI in the acute phase (i.e., within 42 days). A total of 113 concomitant lesions to the cartilage surfaces were observed in 75 of the 176 knees (43%). These were all located on the lateral femoral condyle (*n*=49), patella (*n*=56), or trochlea femur (*n*=8) and consisted of a total of 69 purely chondral lesions, 34 osteochondral fractures, and 10 impaction fractures as visualized on MRI or during surgery (Fig. 5).

There was no statistically significant difference in risk of injury to the cartilage surfaces (*P* = 0.231) with LPD during sports (39%), compared to other activities (48%) or between males (44%) and females (41%, *P* = 0.696). However, males sustained such injuries during sporting activities more often than females (61 vs. 31%, *P* = 0.012). Individuals with injury to the cartilage surfaces were older than those without (median age 18 years [IQR, 15–25] and 16 years [IQR, 14–19], *P* = 0.004).

The subset of the potentially repairable osteochondral fractures showed essentially the same age and gender distribution (median age females 16 years [IQR, 13–22] and males 18 years [IQR 15–23], *P* = 0.478). Thirty-two knees (18% of all LPD that were examined in the sub-acute phase) had osteochondral fractures (13 on the lateral femoral condyle, 17 on the patella, and two on both). The risk of osteochondral fracture was not statistically significantly different for LPD as a sports injury compared to during other activities (14 vs. 24%, *P* = 0.080), nor between males and females (16 vs. 21%, *P* = 0.405). Contrary to any injury to the chondral surfaces, males did not get osteochondral fractures during sports statistically significantly more often than females (53 vs. 27%, *P* = 0.131).

Twenty-two knees with osteochondral injury were subject to radiography prior to MRI. Skeletal injury was suspected in 13 of these (59%). Of the 184 knees with LPD, 60% were correctly diagnosed at the initial visit. Twenty-two percent were diagnosed as either crucial ligament, collateral ligament, or meniscus injury, separately or in combination. The remaining were diagnosed as either distortion or contusion.

## Discussion

The major findings of this study, with a comprehensive consecutive inclusion due to a clinical routine of sub-acute MRI of all knee-injuries in the catchment area, were an overall annual cumulative incidence of 14 primary LPD per 100,000 persons with significantly higher incidences among males compared to females. The incidence of primary LPD was highest at ages 13–15 years for both boys and girls. Two out of five knees with primary LPD sustained concomitant injury to chondral surfaces and two out of five of these were osteochondral fractures with a potential need of assessment for early surgery.

The results confirm that primary LPD commonly occurs during sports in a general as well as a pediatric population [1, 20–22, 24, 29], but with a gender difference not shown before. The most frequent sport, both for females and males as earlier shown in the Swedish population [1], was soccer, followed by gymnastics. Among US high school athletes, Mitchell et al. [30] reported the highest risk of patellar instability injuries per activity exposure in girls’ gymnastics followed by boys’ football, boys’ wrestling, and girls’ soccer, with a generally higher risk of injury during competition than during practice.

Athletic activity, assumedly with higher degrees of impact force compared to leisure activity, could increase the risk of chondral or osteochondral lesions; however, these injuries were not more common during sports than other activity. Among males, the proportion of cartilage injuries during sports was twice as high compared to females, reflecting the sex-dependent pattern of the eliciting activity [30].

The overall risk of a contralateral LPD during the study period appeared to be much higher for those aged 15 years or younger compared to those aged 16 years or older when acquiring the first injury. The small number precludes conclusions, but the observation is similar to the findings by Gravesen et al. [25]. It is fair to assume that a greater proportion of the youngest dislocators show more anatomical risk factors (e.g. trochlear dysplasia, greater sulcus angle, patella alta, and elevated tibial tubercle-trochlear groove [TT-TG] distance) than older first-time dislocators [31, 32].

The reported annual incidence of first-time LPD in the population varies widely, between two and 69 per 100,000 in earlier studies [21–26, 29]. For the paediatric population, reported incidences in different age groups vary between 32 and 120 per 100,000 [1, 20]. It should be noted that samples from several reports were selected from specified populations (private health plan [21, 29], military personnel [22, 23], pediatric [1, 20]) or derived from registries [24–26] and therefore may not be representative of a general population or may have less degree of diagnostic control. The only previous report from a consecutive clinical series included 125 primary LPD in the Kaiser Foundation Health Plan where annual incidences of 5.8 and 29 per 100,000 members for all members and members aged ten to 17 years, respectively [21, 29], were found. Incidences reported in our study were twice as high, which is likely explained by the difference in diagnostic precision (i.e., clinical diagnosis vs. MRI).

On the other hand, two recent registry-based studies have reported higher annual incidences of primary LPD [25, 26]. One of these showed significant fluctuations in incidence over time, which may reflect the tendency among doctors of diagnosing LPD, lacking confirmation by MRI [25]. Peak incidences of primary LPD have uniformly been shown to occur in the paediatric population, which agree well with our results [1].

Except for studies of military populations [22, 23], we report the largest fraction of male dislocators (59%), particularly among boys in ages between 16 and 18 years (73%). We hypothesize that a tendency to still consider LPD a female diagnosis may exist and, thus, the diagnosis of LPD in a twisted knee of an adolescent male does not come to mind as easily as other differential diagnoses. There was a tendency towards higher incidences for girls in the youngest dislocators, and a higher male incidence over the age of around 14 which is in concordance with the report of Sanders et al. [26]. Earlier studies have shown equal distribution of male and female first-time dislocators, ranging between 46 and 53 [21, 24–26, 29]. Askenberger et al. [1] reported a somewhat larger male fraction (56%) in a young population aged nine to 14 years, but with twice as many boys than girls between 12 and 14 years. In contrast, Nietosvaara et al. [20] reported only 32% boys in the age range of nine to 15 years.

Using arthroscopy and/or MRI for assessment, previous studies have reported cartilage injuries in 34 to 95% of the knees after LPD [12, 13, 16, 20, 33–40]. Agreement between arthroscopy and MRI in diagnosing cartilage lesions grade 3 and 4 has been reported to be good to very good in primary LPD, but poorer for milder injuries [41]. We report cartilage injury in 43% of the knees as detected on MRI or in some cases an urgent arthroscopy. The relatively low rate compared to some other reports may be due to a broader inclusion of patients. Eighteen percent of LPD suffered an osteochondral fracture, less than half of these during sports, in line with the 12% of knees with osteochondral fracture reported by Askenberger et al. [1]. The location of the osteochondral fractures with 50% on the patella, 46% on lateral femur and 4% on both, was slightly different than recently reported by Uimonen et al. [42]. They reported 99 such injuries after primary patellar dislocation and 35 after recurrent dislocation in a similar population sample of both pediatric and adult patients with a greater proportion of injuries on the patella (62%). Although fairly consistent with their subgroup analysis of male dislocators who showed a more even distribution of the injuries between patella and femur, no gender-dependence could be detected on the rate of chondral injuries in contrast to a recent report from Zheng et al. [43]. The inclusion in the current study was not limited to the clinical diagnosis of LPD, and more male patellar dislocations otherwise maybe misdiagnosed as other knee-injuries were reported. Thus, gender may play a role in the location of osteochondral fracture, even though our sample is too small for comparisons.

As osteochondral injuries have been suggested to lead to progressive cartilage degeneration and osteoarthritis, early and correct diagnosis of LPD and concomitant injuries is imperative to allow timely surgical repair when possible [44, 45]. In their study of paediatric knee injuries, Askenberger et al. [1] showed that the diagnosis of LPD was not clear in 27% of the cases before an MRI was performed. Sanders et al. [7] evaluated risk factors of patellofemoral arthritis after 609 first-time LPD, where an osteochondral fracture on radiographs or MRI showed the greatest hazard ratio, twice as high as recurrent dislocations. In our study, more than four out of ten osteochondral fractures were not suspected from plain radiographs, highlighting the significant risk of misdiagnosing not only the LPD but also concomitant cartilage injuries.

### Limitations

This study has certain limitations. It is likely that not all individuals with acute knee injury sought medical attention and some of those attended in primary care were probably not referred to the orthopedic department. These limitations would suggest possible minor underestimates regarding reported incidences of LPD and may be an overestimation of cartilage injuries as individuals with less traumatic LPD may be more prone not to seek medical attention.

Major strengths of this study were the prospective consecutive inclusion of all knee injuries with traumatic haemarthrosis, the well-defined catchment area of a hospital serving a complete adult and pediatric population, and the subsequent examination of all knees with MRI, regardless of initial clinical diagnosis. Moreover, the surgical records at the only two hospitals offering arthroscopic surgery for the relevant population where all surgeries are noted with the patients’ personal identification number were scrutinized for missed injuries and are thought to be comprehensive.

## Conclusions

The annual incidence of first-time patellar dislocation was found to be 14 per 100,000 individuals with the highest incidence found among those aged 13–15 years. Primary LPD was more common among males and was sustained during sports activity in 55% of the cases. Associated injuries to the chondral surfaces should be expected in 43% of knees with primary LPD where 18% represent osteochondral fractures.
